# Alterations of NoGo P300 ERP in schizophrenia in social setting: a hyperscanning study

**DOI:** 10.1038/s41398-025-03481-6

**Published:** 2025-08-07

**Authors:** Máté Fullajtár, Brigitta Kakuszi, István Bitter, Pál Czobor

**Affiliations:** Semmelweis University, Department of Psychiatry and Psychotherapy, Budapest, Hungary

**Keywords:** Schizophrenia, Physiology

## Abstract

Although patients with schizophrenia exhibit profound deficits in social cognition, studies into the neurobiological background of these deficits examined individuals in isolation, in single-person settings. We investigated the neurobiological basis of social cognitive deficits in a social setting, applying a novel approach using EEG-hyperscanning. Eighty subjects were included in the analyses, 49 healthy controls (HC) and 31 patients with schizophrenia. We recorded high-density EEG from pairs of participants, where one (the observer) watched their own screen while the other (the actor) actively performed a Go/NoGo task. The task was administered twice, with the participants switching roles. We focused on investigating the P300 event-related potential from the observer condition. The PANSS scale was used to characterize psychopathology. The Reading the Mind in the Eyes Test and the d-prime index were applied to characterize mentalization and signal detection ability. We found that patients with schizophrenia showed significant P300 reduction compared to the HC group at the first task exposure. They, however, exhibited augmented P300 with the repeated exposure, while HCs manifested a decrease. More severe positive symptoms were associated with larger P300 at both task-exposures. Moreover, poorer mentalization and signal detection performance were associated with decreased P300. Our findings provide evidence that P300 alterations in schizophrenia can be detected in social setting. The opposite changes in the two groups may be due to disparate reasons: habituation in HCs, whereas the alterations in patients may result from various factors, including deficient habituation and an aberrant processing of stimulus salience in a social setting.

## Introduction

Schizophrenia is a chronic psychiatric disorder that can be defined as a syndrome [1, 2]. Based on clinical manifestations, positive (e.g., delusions), negative (e.g., impoverishment of emotional life) and cognitive symptoms can be distinguished [1, 2]. Importantly, the literature has also highlighted a basic impairment of social cognition in patients with schizophrenia [3]. Social cognition is an important determinant of functional disease outcome, since it acts as a mediator between neurocognitive functions and everyday functioning [4, 5].

Deficits in social cognition and interactions in schizophrenia can be detected in many areas of the patients’ daily lives (e.g., maintenance of social relationships and independent living) [6, 7]. Based on the literature, four basic areas of social cognition are considered impaired in schizophrenia, including emotional processing, social perception, attribution style, and theory of mind (ToM) [8]. However, due to design and technological limitations, past studies of the neurobiological basis of these impairments have mostly been based in single-subject task settings [9, 10], despite the fact that social interactions are an important part of social behavior.

Recently, a novel technology, termed hyperscanning has become available to characterize the neurobiological background of deficits in social settings [11, 12]. Specifically, hyperscanning offers an insight into the neurobiological background of the interaction of two or more people through their actual physical presence in real world settings [11, 13]. There has been a range of studies which adopted the hyperscanning approach to examine social interactions between people with close personal relationships [14]. The most frequently used hyperscanning approaches rely on electroencephalography (EEG), functional magnetic resonance imaging (fMRI), magnetoencephalography (MEG), and functional near infra-red spectroscopy (fNIRS) [11, 14], which all have various advantages and disadvantages. A principal advantage of EEG-based studies is that, besides their good temporal resolution, they can be applied in real-life conditions to investigate the neurobiological processes underlying behavior in social settings [11, 12]. Besides the investigation of resting activity, EEG-based hyperscanning approaches often rely on the studies of event-related potentials (ERPs). Event-related potentials (ERPs) represent the brain’s transient responses to specific external or internal stimuli, or events of specific significance such as the lack of an expected stimulus, which generates a specific response, the P300 ERP in our study. ERPs provide a safe and noninvasive approach to study psychophysiological correlates of mental processes.

The association between the P300 ERP and schizophrenia symptoms has been demonstrated in a number of studies and summarized in various meta-analyses [15, 16]. The NoGo P300 component, which was the focus of the current study, has a frontocentral maximum and is considered to be modulated by several factors, including habituation, priming to the particular situation, and an abnormal evaluation of the salience of the presented stimuli [17]. These factors are particularly relevant with respect to the symptom presentation in mental disorders, which makes it worthwhile to examine this ERP component in clinical studies [18]. A large body of empirical evidence has accumulated indicating that P300 can serve as a transdiagnostic, prognostic neuromarker for schizophrenia spectrum, bipolar disorder, obsessive compulsive disorder [19, 20]. For example, based on a large data set from The North American Longitudinal Follow-Up Study, the P300 proved to be reliable predictor of conversion to psychosis in individuals in high risk, psychosis prone subjects [21].

While evidence indicates that patients with schizophrenia manifest reduced P300 amplitude [22–24], important unanswered questions remain regarding the P300 alterations reported in these disorders. For example, it is noteworthy that the information regarding P300 deficits in schizophrenia has been gathered in single-subject studies, despite the fact that the disease is viewed as a condition with impaired social cognition. Thus, prior studies cannot inform whether alterations detected in single subject studies can also be observed in social settings. Prior studies also remain equivocal about the relationship between schizophrenia symptoms and P300, with some of the studies showing a reduction, while others reporting no change or even an enhancement [25, 26].

To fill these knowledge gaps in the literature, our aim was to characterize the neurobiological background of P300 alterations in schizophrenia in a social setting during a Go/NoGo task paradigm using an observer-actor test situation. We applied a Go/NoGo task situation because it allows for the investigation of the neurobiological underpinnings of deficits in behavioral response inhibition, and impairments of executive functions, which are often manifested in schizophrenia, especially in social settings [27]. The NoGo P300 component is considered to be modulated by several factors, including habituation, priming to the particular situation, and an abnormal evaluation of the salience of the presented stimuli [17]. These factors are particularly relevant with respect to the symptom presentation in schizophrenia, which makes it worthwhile to examine this ERP component in clinical studies [18].

We also investigated the impact of mentalization and signal detection ability of participating individuals since these factors can influence behavioral performance in a social situation. Prior literature has shown a mentalization deficit in patients with schizophrenia based on the RMET test [28, 29]. In addition to mentalization, the signal detection ability of individuals participating in social interactions is particularly important since it can interfere with the decision-making performance. Signal detection theory can offer an insight into how individuals make decisions based on the evidence they can receive. The signal detection ability of an individual can be measured by the d-prime index, which characterizes the observer’s ability to select the right stimuli while avoiding the wrong ones [30]. Therefore, besides psychopathology, we included these two factors (mentalization and signal detection ability) as predictors in our analysis.

## Methods

### Participants

The current exploratory study included 84 participants, 32 inpatients with the diagnosis of schizophrenia (SZ, 16 pairs) and 52 healthy control (HC, 26 pairs) between the age of 18 and 65 years. The study was a single-center trial, enrolling in-patients admitted to the Department of Psychiatry and Psychotherapy, Semmelweis University. In one subject in the SZ, and three subjects in the HC group the EEG recordings were not evaluable due to artifacts, therefore these subjects were not included in the analyses. Accordingly, for the analysis set, the patient group consisted of 24 (77%) males and 7 (23%) females; the analogous numbers for controls were: 19 (39%) males and 30 (61%) females. The mean age in the patient and the control group was 35.6 years (SD = 12.5) and 30.2 years (SD = 9.5), respectively. Patients with schizophrenia were diagnosed on the basis of DSM-5 [31]. The exclusion criteria for both groups included the presence of known severe neurological or somatic history, such as head injury, or alcohol or drug use before the EEG examination. Participating patients came from an inpatient setting, where no access to drug and alcohol use was permitted.

In the control group, the inclusion criteria were: the lack of psychiatric illness, and the score on the 90-item SCL-90R symptom checklist [32] below the clinical range, established for the general population in Hungary [33]. Patients with the diagnosis of schizophrenia received pharmacotherapy. Antipsychotic treatment during the study was characterized by the chlorpromazine (CPZ) equivalent dose [34]. Participants provided written informed consent according to the protocol in compliance with the Declaration of Helsinki and approved by the local ethics committee (Ethics Committee of Semmelweis University, #116481/AOPSI/2018).

### Measurement of psychopathology

Symptoms of schizophrenia were measured by the Positive and Negative Syndrome Scale (PANSS), which consists of 30 items, positive and negative symptom subscales with 7 items each with 7 items, respectively, representing the positive and negative symptom subscales, and 16 items representing the general psychopathology subscale [35]. Items were scored between 1 through 7, with a total positive and negative subscale score of 7 through 49; a general psychopathology subscale score of 16 through 112; and total score ranging between 30 and 210. The PANSS scale can be used for a detailed characterization of psychopathology based on a 5-dimensional factor-model. Specifically, the most commonly applied PANSS factors are positive symptoms, negative symptoms, disorganized thought, uncontrolled hostility/excitement and depression/anxiety [36, 37]. The PANSS was administered as a semi-structured clinical interview by a clinician trained in the use of the instrument.

### Stimulus paradigm

Two participants were examined simultaneously in pairs, sitting next to each other in a dimly lit room, both in front of a separate monitor, seated approximately 100 cm from the monitors. We applied a Go/NoGo paradigm as the stimulus task situation, using pictures from The International Affective Picture System (IAPS) [38, 39] representing negative, positive, or neutral emotional valences. The pictures were displayed using the Presentation 13.0 software (Neurobehavioral Systems, Inc. Albany, California, USA).

Participants were instructed to respond to each picture by pressing a key as soon as possible (Go condition), unless the picture was presented immediately before (NoGo condition). The stimulus cycle time was 1400 ms. Each picture was displayed for 800 ms, followed by a 600 ms inter-stimulus interval. In total, 480 stimuli were presented to each participant, divided into two blocks, with 240 images in each block. The probability of Go and NoGo task was 85 and 15%, respectively. During the stimulus presentation both participants were shown the same pictures, with one of the participants placed in an observer situation, while the other was in an actor situation. The actors actively performed the task. The observers watched their screen as the actors performed the task. Participants’ roles were then reversed, so that all participants were in both situations. The order of the two conditions was randomized across the various pairs. In each stimulus cycle, a red or blue dot appeared on the screen 1200 ms after stimulus presentation to indicate a correct or incorrect response, respectively.

### EEG recording and pre-processing

EEG data were obtained by using two 256-channel active electrode amplifier system (Biosemi Inc, Amsterdam, Netherlands) at a sampling rate of 512 Hz. The two amplifier systems were connected in daisy-chain mode to allow for the recording of EEG signals from 128 channels from each of the two individuals participating in recording session [40]. The signal was bandpass filtered in the range of 0.5–100 Hz using the average reference. Electro-Magnetic Source Signal Imaging Suit (EMSE Suit) software version 5.1 was used to pre-screen electrophysiological data. The raw EEG data were filtered as follows: an IIR Butterworth filter (0.5–70 Hz) was used as a band-pass filter and a Parks-McClellan stop-band notch filter (48–52 Hz) was applied to omit power line noise. After correction for eye movement artifacts and filtering for baseline fluctuations using a third-order polynomial filter, the recordings were manually reviewed off-line. Subsequently, the EEG data were analyzed using Statistical Analysis System (SAS) version 9.4. As requested, this information has been added to the revised manuscript.

### Performance on the RMET test and behavioral measures

During the RMET testing, the subject is presented with a series of 36 pictures of the eye-region of the face of different people and is asked to choose which of four words best describes what the person in the images is thinking or feeling. All participants completed the RMET test before stimulus presentation, with the task applied off-line with no time limitation. To characterize performance on the RMET, we used the rate of correct answers to the 36 picture presentations during the test, yielding a score between 0 and 1 [41, 42].

Speed of responding (reaction time) was characterized by measures of central tendency (median, mean) for each participant when placed in the actor situation. We also examined the proportion of omission and commission errors. Similar to prior literature [43], signal detection ability was characterized by the d-prime index, which captures information from the omission and commission errors into one single measure. Specifically, d-prime was computed as the difference between z-transformed value of the hit rate (proportion of correct responses) and the z-transformed value of commission error rate. Higher values of d-prime indicate better performance. The d-prime can take positive and negative values, with negative d-prime estimates indicating that the subject is sensitive to the signal, but tends to produce incorrect responses. Thus, in our study, the reaction time and signal detection indices were derived from the Go/NoGo task, which was administered during the EEG session, while performance on the RMET was derived from the off-line administration of the RMET task.

### Statistical analysis

#### RMET and behavioral measures

Analysis of covariance was applied to examine the group differences in terms of the performance on the RMET and behavioral measures. Reaction time, rates of commission and omission errors, d-prime and the accuracy on the RMET were used as dependent variables while diagnostic group membership was applied as independent variable. Age and gender were used as covariates.

#### ERP measures

Based on literature, the definition of frontal P3 component time-window included the post-stimulus epoch of 300–450 ms [44]. Similar to our earlier work [45], the statistical analyses were based on random-regression hierarchical linear modeling (HLM) [46, 47]. All statistical tests were two-sided. In the analysis of group difference, repeated measurements of the ERP amplitude (in microvolts in the frontal NoGo P3 component time-window) in the frontal regions served as dependent variable. For the P3 ERP, the region of interest included electrode Fz and the adjacent electrodes surrounding Fz in the BioSemi recording system, i.e., the three mid-anterior electrodes in the International 10–20 System, and the midline and lateral electrodes between them (the latter, labelled as C22, C11, and C24, respectively, in the BioSemi-layout). Study group (SZ vs. HC) and exposure (first vs. repeated exposure to the task) and their interaction were used as the principal independent variables of interest.

Time (sampling point in the component window, relative to stimulus onset) was included in the analysis as a within-subject factor, representing the series of microvolt values in the P3 component time-window. Exposure was used as an additional within-subject factor to indicate whether the particular P300 response was obtained from the first as opposed to the second exposure to the task. In the HLM model, a first-order autoregressive moving average correlation matrix was applied to describe the time dependent correlation among the sampling points. Age and gender served as covariates. Correction for multiple testing was performed using the False Discovery Rate (FDR) procedure [48]. For the analyses of the association of P300 amplitude with the behavioral measures, performance on the RMET test and signal detection, the above HLM model was expanded by including these measures as covariates. In the analyses of psychopathological and clinical variables, the group effect was omitted from the HLM model.

## Results

### Demographics, descriptive statistics

Descriptive statistics for study subjects are presented in Table 1. As shown by the Table, patients with schizophrenia had a somewhat higher age (approx. 5 years), the difference reached the level of statistical significance. The two groups also differed in terms of gender distribution and level of education. Specifically, in the SZ group males were overrepresented, while the HC group had an approximately balanced gender distribution. With respect to education the proportion of subjects with medium and higher education was lower in the schizophrenia sample. As shown by the total score on the PANSS scale, our sample manifested a high level of psychopathology with a mean total score of 102.4 (SD = 21.4). Patients had an average duration of illness of 9.1 years (SD = 9.1). Antipsychotic therapy was characterized by the chlorpromazine equivalent dose (CPZ); the average CPZ dose was 618.3 (SD = 351.7). A total of 32(64%) subjects with schizophrenia had concomitant benzodiazepine medication at the time of EEG recordings. As indicated by the subscales scores in Table 1, the severity of psychopathology was similar between positive and negative symptoms and general psychopathology. Similar to the subscale scores, the severity on the positive and negative factors, defined according to Marder et al.’s, were comparable.

**Table 1:** Basic demographic and clinical characteristics of the study sample^a^.

Characteristics	HC	SZ		
Categorical variables N (%)	*N* = 49^b^	*N* = 31^c^	Chi^2^	p
Demographic				
Male/Female, N (%)	19/30 (39/61)	24/7 (77/23)	11.41	<0.01
Education level (n1/n2)^d^	34/15	29/2	22.97	<0.01

Continuous variables:	Mean (SD)		F	p
Age (years)	30.2 (9.5)	35.6 (12.5)	4.86	<0.03
PANSS total score	—^e^	102.4 (21.4)	—^e^	—
Positive subscale score (sum of 7 items)	—	24.0 (6.5)	—	—
Negative subscale score (sum of 7 items)	—	25.4 (5.9)	—	—
General psychopathology subscale score (sum of 16 items)	—	53.0 (12.0)	—	—
CPZ—chlorpromazine equivalent daily dosage	—	618.3 (351.7)	—	—
Disease duration (years)	—	9.1 (9.1)	—	—
Reaction time (ms)	374.6 (125.8)	562.6 (119.0)	570.46	<0.0001
Commission error (%)	11.9 (12.5)	42.6 (22.1)	142.78	<0.0001
Omission error (%)	2.2 (5.5)	16.7 (12.3)	114.96	<0.0001
Signal detection (d-prime)	2.58 (0.87)	0.70 (2.67)	43.33	<0.0001
RMET test (proportion correct)	0.71 (0.14)	0.54 (0.14)	26.10	<0.0001

^a^Chi-square test for categorical, ANOVA for continuous variables

^b^26 pairs of HC were examined, the EEG was not evaluable in 3 subjects due to artifacts.

^c^16 pairs of SZ were examined, the EEG was not evaluable in 1 subject due to artifacts.

^d^n1, without college degree; n2, College/University degree.

^e^Not applicable.

### Group differences in behavioral measures and performance on the RMET test

As depicted in Table 1, significantly higher rate of omission and commission errors was observed in patients with schizophrenia as compared to the controls (omissions: F = 114.96, df = 1,73, *p* = <0.0001; commissions: F = 142.78, df = 1,73, *p* = <0.0001). In terms of overall signal detection ability, as measured by the d-prime, the patient group evidenced significantly lower scores compared to the controls (F = 43.33, df = 1,73, *p* = <0.0001). Additionally, patients with schizophrenia also exhibited a significantly longer reaction time to the stimuli (F = 570.46, df = 1,73, *p* = <0.0001). Furthermore, with respect to the RMET test, the patient group had a significantly lower accuracy relative to the HC group (F = 26.10, df = 1,73, *p* = <0.0001; patient mean/SD = 0.55/0.14; control mean/SD = 0.71/0.14;).

### Electrophysiological findings

#### Group differences in P300 amplitude

The primary HLM analysis with two factors, which included group (SZ, HC), exposure (1^st^, 2^nd^ exposure to the task) and their interaction, showed a significant interaction between these two factors (F = 63.70; df = 1,77; *p* < 0.0001) with no main effect for group (F = 0.17; df = 1,77; *p* = 0.6867) or exposure (F = 0.58; df = 1,77; *p* = 0.4475). Post-hoc analysis for the interaction revealed a significant group difference (t = 5.87; df = 1,77; *p* < 0.0001) at 1^st^ exposure, indicating that in patients with schizophrenia the P300 amplitude manifested a significant reduction compared to the HC group. We note that in our statistical analysis of the P300 amplitude and in the post-hoc analyses, we found highly statistically significant differences, which remain significant after correction for multiple testing using the FDR procedure [48].

**Fig. 1:**
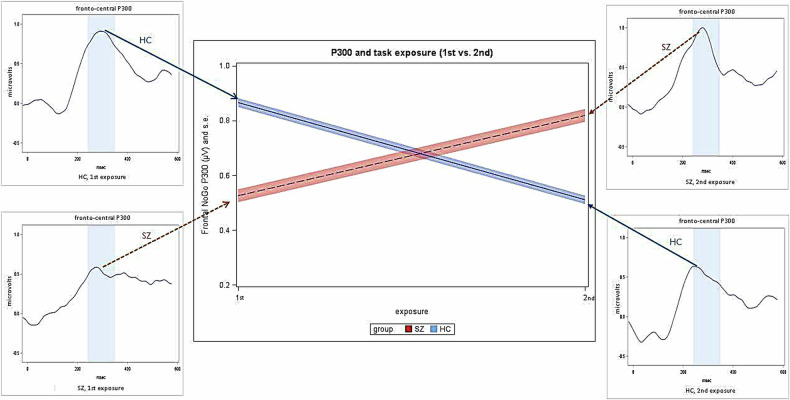
Grand average ERPs for the control (HC) and schizophrenia (SZ) groups. The left and right panels in the figure show the grand average ERPs for the control (HC) and schizophrenia (SZ) groups, respectively, at the 1st (left) and 2sd exposure (right). The middle part of the figure depicts the mean and standard error (shaded area) of the amplitude of the NoGo P300 component, as estimated from the HLM analysis. Patients with schizophrenia, when placed in the observer situation (red line) manifested a significant (*p* < 0.05) amplitude reduction compared to the HC group (blue line) at 1st exposure. The temporal sequence (repetition) of the stimulus presentation had a differential impact on the P300 amplitude in the SZ and HC group: in the patients, a significant augmentation in P300 amplitude occurred in the repeated test situation compared to the first exposure (1st). By contrast, the P300 amplitude in the control subjects showed a significant decrease in the repeated test situation (2nd).

Furthermore, the temporal sequence of the stimulus presentation had a differential impact on the P300 amplitude in the SZ and HC group. In particular, in the control subjects a significant decrease (t = 7.8; df = 1,77; *p* < 0.0001) in response to the repeated presentation of the stimuli occurred as compared to the initial exposure to the task. By contrast, patients with schizophrenia exhibited a significant increase with the repeated exposure (t = 4.36; df = 1,77; *p* < 0.0001). Fig. 1 depicts the grand average ERPs in each of the two groups at both exposures, and illustrates the profile plots for the differential changes in the P300 in the two groups as a result of repeated exposure.

#### Association of P300 with social cognitive ability and signal detection performance

**Fig. 2:**
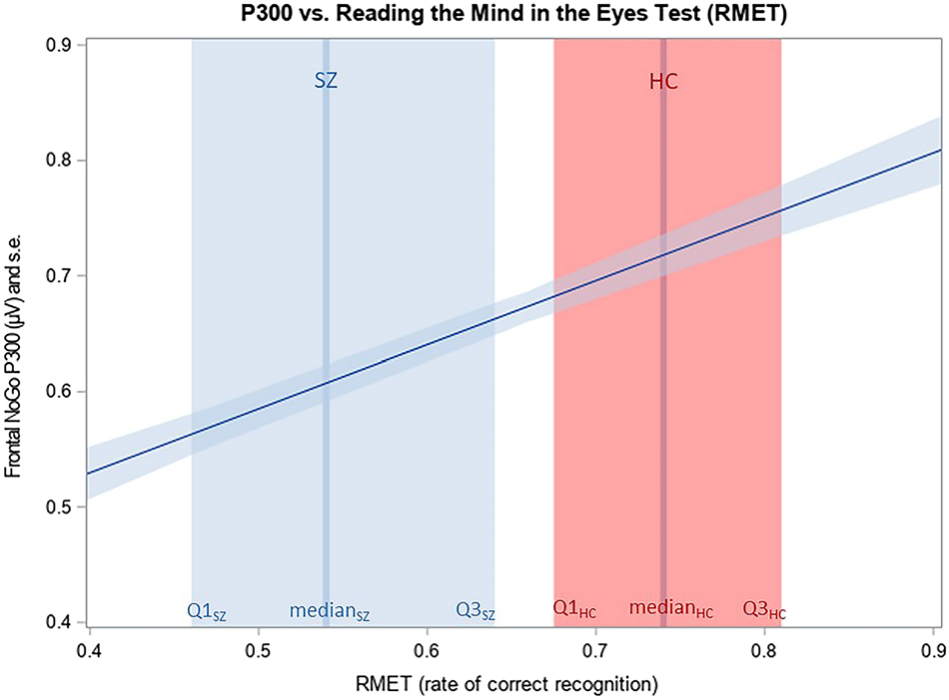
Relationship between P300 and social cognitive ability, as measured by the performance on the RMET test. The horizontal axis shows the correct recognition rate on the RMET, and the vertical axis shows the P300 amplitude (shaded area = standard error). Patients with schizophrenia achieved significantly lower scores on the RMET (median and interquartile range (Q1–Q3) indicated in vertical blue column) as compared to healthy control group (median and interquartile range (Q1–Q3) indicated in vertical red column). In both groups, poorer performance on the RMET was associated with lower P300 amplitude (blue line), with no group difference in the relationship.

The HLM analysis of the association between P300 and social cognitive ability, as measured by the performance on the RMET, indicated a significant main effect for the correct recognition rate on the P300 amplitude (F = 54.49; df = 1,77; *p* < 0.0001). The interaction of correct recognition rate on the RMET with diagnostic group was not significant (F = 1.58; df = 1,77; *p* = 0.2130). Post-hoc analysis of the main effect showed that poorer performance on the RMET was associated with lower P300 amplitude in both groups. Fig. 2 depicts the association using the LSmeans estimates from the HLM model for the P300 amplitude at various levels of RMET performance. The regression relationship is shown across the two study groups since the interaction between RMET performance and diagnostic group was not significant.

**Fig. 3:**
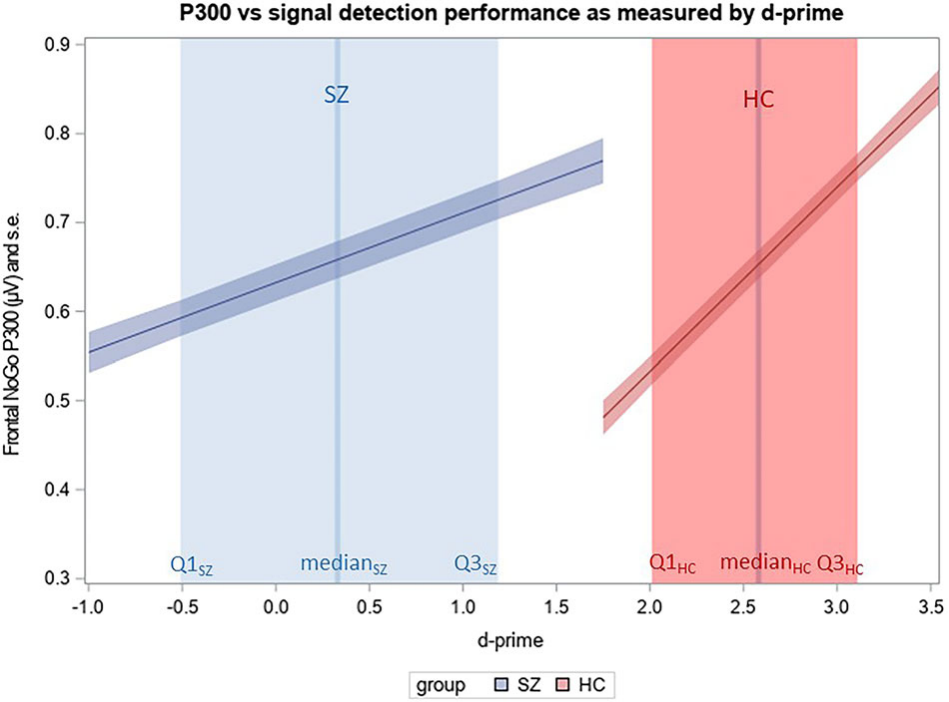
Relationship between P300 and signal detection performance as measured by d-prime index. The horizontal axis shows the d-prime index (higher values indicated better signal detection), and the vertical axis shows the P300 amplitude (shaded area = standard error). Patients with schizophrenia (SZ) achieved significantly lower scores on the d-prime (median and interquartile range (Q1–Q3) indicated in vertical blue column) as compared to healthy control (HC) group (median and interquartile range (Q1–Q3) indicated in vertical red column). In both groups, poorer performance as measured by d-prime was associated with lower P300 amplitude (blue line). Poorer signal detection ability was related to lower P300 (blue and red line) in both groups, with closer association in the HC group (red column) as soon by a significant (*p* < 0.05) interaction in HLM analysis.

The analysis of the relationship between P300 amplitude and signal detection ability included d-prime as an independent variable in the HLM model. Results of the analysis showed a significant main effect for d-prime (F = 139.11; df = 1,77; *p* < 0.0001) as well as a significant interaction for d-prime with the diagnostic group (F = 35.53; df = 1,77; *p* < 0.0001). Post-hoc analysis of the main effect showed that in both groups better signal detection ability, as indexed by higher values on the d-prime, was related to larger P300 amplitude. Moreover, the analysis of the interaction effect indicated a less pronounced association in the SZ as compared to the HC group. Fig. 3 shows the association in the two groups using the LSmeans estimates from the HLM model. The regression relationship is shown separately for the two study groups as the interaction between RMET performance and diagnostic group obtained significance.

#### Association of P300 amplitude with psychopathology

**Fig. 4:**
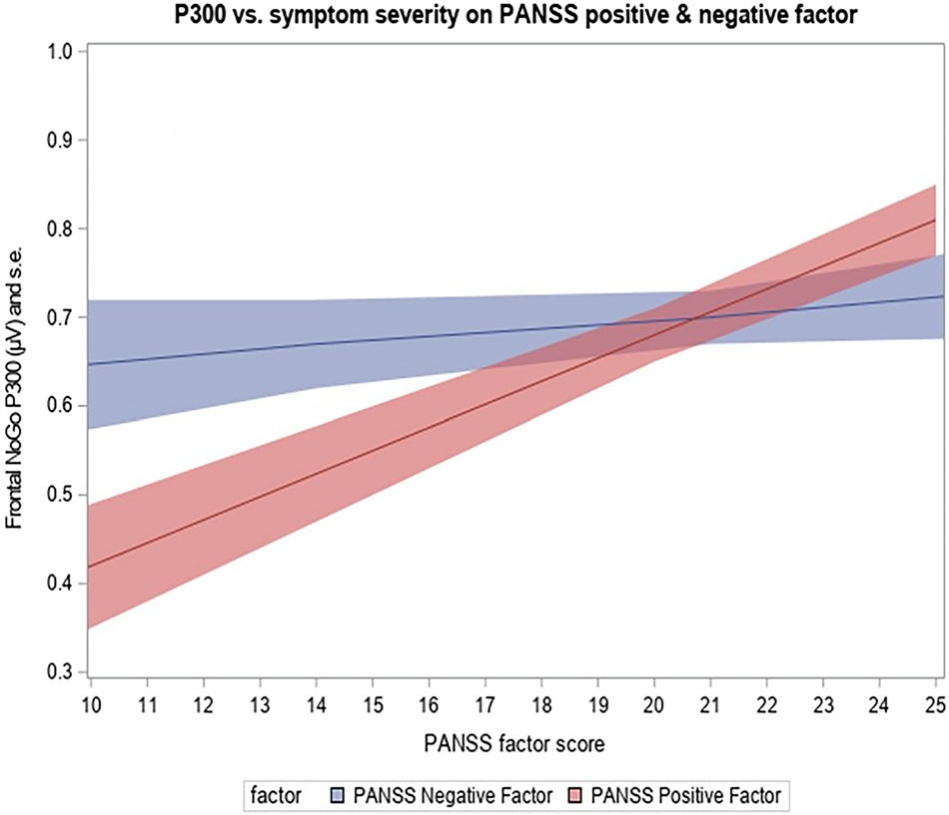
Association of the P300 amplitude with the positive (red) and negative (blue) symptoms of schizophrenia. The P300 amplitude (mean shaded area = standard errors) exhibited significant (*p* < 0.05) positive association with the total score of the PANSS positive factor regardless of the exposure, while no association between P300 amplitude, and the factor score on the negative symptoms was present.

We investigated whether P300 was associated with the severity of positive and negative symptoms of schizophrenia, as measured, respectively, by the positive and negative factors of the PANSS scale. Our results showed that the P300 amplitude had a significant overall positive association (F = 21.08; df = 1,29; *p* < 0.0001) with the total score of the PANSS positive factor regardless of the exposure. The results obtained significance (*p* < 0.0001) at both task exposures. With respect to negative symptoms, our results revealed no overall association between P300 amplitude, and the factor score on the negative symptoms (F = 0.51; df = 1,29; *p* = 0.4828), with no relationship present at either of the two exposures (*p* > 0.1). The findings for the association of P300 amplitude with positive and negative symptoms, respectively, as shown in Fig. 4.

#### P300 amplitude and demographic and clinical characteristics

The analyses conducted in the two study groups with respect to the potential influence of gender on the P300 amplitude showed no significant main effect of gender (F = 0.96; df = 1,79; *p* = 0.3302) or an interaction between gender and study group (F = 2.57; df = 1,79; *p* = 0.1134). With respect to age, we found no effect (F = 1.09; df = 1,79; *p* = 0.3007). The interaction, however, was statistically significant (F = 21.30; df = 1,79; *p* < 0.0001), with a positive association between P300 amplitude and age in the SZ group, and an inverse relationship in the HC group.

We respect to chronicity, our results indicated a positive relationship between P300 amplitude and duration of illness (F = 31.04; df = 1,29; *p* < 0.0001): patients with longer duration of illness had higher amplitude as compared to patients with shorter illness duration. We note that the association between P300 amplitude and duration of illness was independent of the age, since introduction of age as a covariate in the analysis did not change the association. Patients in our study received antipsychotic treatment during the study, which was characterized by the CPZ equivalent dose and benzodiazepine (BZD) administration. We found no association between P300 amplitude and BZD administration. TheP300 amplitude exhibited a significant positive association with the CPZ dose in both test situations (F = 7.46; df = 1,29; *p* = 0.0108). Specifically, the higher the CPZ equivalent dose, the greater the P300 was in the patients with schizophrenia.

## Discussion

Our study investigated the neurobiological background of social cognition in a social situation using EEG hyperscanning. Specifically, we focused on the P300 ERP in an observer-actor setting. The novel technology that we used, i.e., the EEG hyperscanning was developed to investigate the neurobiological background of deficits in social contexts [10, 11]. As highlighted in the literature [10, 12], it offers an insight into the neurobiological background of the behaviors of two or more people in various social scenarios, starting from the actual joint physical presence to complex social interactions [10, 12, 49]. In our study, we applied EEG hyperscanning in a simple social context (i.e. observer/actor).

At the behavioral level, our results showed a significant increase in reaction times in patients with schizophrenia in the Go/NoGo task situation compared to the control group. Similar to the increased reaction times, a significant increase in omission and commission errors was present in the SZ group compared to HC when participants were investigated in the actor situation. Hence, our results indicate that, similar to single-subject task situations [8, 9], deficits in behavioral measures also occur in a simple social setting among patients with schizophrenia.

We also found that patients with schizophrenia achieved significantly lower scores on the RMET test than the HC group. Furthermore, in both groups poorer performance on the RMET was associated with smaller P300, with no group difference in the relationship. We also observed significantly lower d-prime values in patients with schizophrenia than in the controls. Additionally, we found that poorer signal detection ability was related to smaller P300 in both groups, with closer association in the HC group as shown by the significant group interaction. Overall, our results are consistent with the literature, which indicated that patients with schizophrenia achieve significantly lower scores on the RMET test as compared to the healthy control subjects. Moreover, we observed significantly lower d-prime values in patients with schizophrenia than in the controls, similar to prior literature [50–52]. The fact that we found a closer association between signal detection ability and P300 in HC than in patients may be attributable to several factors, including psychopathological symptoms of schizophrenia or to the use of medication, which can modulate the relationship.

Our results also revealed that in patients with schizophrenia the temporal sequence of stimulus presentation played a role in the alterations in the observer situation. Prior exposure to the task elicited a significant effect on the P300 response during the subsequent presentation, which was different in SZ and HC. In the patients, significant augmentation in P300 occurred in the repeated test situation compared to the first exposure. By contrast, the P300 amplitude in the HCs showed a significant decrease in the repeated test situation. These changes in the opposite direction may be due to disparate reasons in the two groups. Specifically, habituation could have occurred in HC, while in patients with schizophrenia the results may be attributable to a mixture of two factors, including a deficient habituation [53] and maladaptive changes through an aberrant processing of stimulus salience [54, 55].

Aberrant salience and abnormal attribution of importance/significance was postulated by Kapur [54] as a major factor behind the psychotic symptoms in schizophrenia. As hypothesized by Kapur, in the affected individuals an abnormally high level of mesostriatal dopamine release would result in an overstimulation of the significance and motivational impact of irrelevant environmental stimuli. This leads to a sense that the world is filled with events of abnormal significance, or has changed in some unusual way (i.e., resulting in the emergence of derealization, and, most importantly, abnormal attributions of importance). According to the recent formulations of the salience theory, increased subcortical dopamine transmission can induce maladaptive changes, substantially altering the perception of the world and leading to the development of abnormal significance attribution, associations (trains of thought), delusions, and hallucinations [56].

The P300 amplitude exhibited significant overall positive association with the PANSS positive factor score, while with respect to negative factors score, our results revealed no overall association. The association of the P300 amplitude with the positive psychopathology in schizophrenia may be related to cognitive impairments [57] and deficits of sensory gating. Sensory gating impairment is theorized to result in an information overload in patients with schizophrenia as a result of the dysfunction in sensory filters. This, in turn, would lead to abnormal experiences, emerging as a secondary consequence. Owing to the afore-mentioned impairments including sensory gating and an aberrant evaluation of salience associated with positive symptoms an increase in task difficulty could occur even in a simple social situation, which in turn could result in a P300 increase [9]. These effects (i.e., sensory gating and aberrant salience) could be further amplified by the effect of priming in our study, which has been postulated in schizophrenia as a factor leading to hyperpriming [58], owing to the abnormal evaluation of stimulus significance. The literature suggests that the mechanism of impairment in sensory gating at the pre-attentive level, as reflected by the lack of P50 attenuation, may underlie the positive symptoms of schizophrenia [59], since the lack of sensory gating may lead to sensory overload. Based on this finding, we can hypothesize that the alterations of P300 that we found in our study may be due to similar mechanism at a later stage of information processing at the attentive level, in association with the social context.

We found that the CPZ dose showed a significant positive association with P300 irrespective of the temporal order of stimulus presentation. Thus, higher CPZ doses are associated with the normalization P300, a relationship which is consistent with the interpretation in the literature that antipsychotic therapy leads to an increase in P300 in schizophrenia [60, 61]. Our results also showed that in patients with schizophrenia, the P300 had a positive association with the illness duration. Thus, it is conceivable that antipsychotic therapy administered during a longer duration of illness may shift the P300 amplitude towards normalization, but this needs to be investigated in further studies.

Patients with schizophrenia in our study had a high level of psychopathological symptoms. Accordingly, they received a high dose of antipsychotic medication, as shown by the CPZ equivalent dose in an inpatient setting throughout the study. Thus, the high level of psychopathological symptoms and the applied medication(s) may have influenced the behavioral and electrophysiological measures. A further limitation is that there was a difference between male-to-female ratios in the HC and SZ group, although the analyses were adjusted for this covariate. Despite these limitations, our study provides novel information on P300 alterations in schizophrenia in a social situation and offers an insight into how these alterations are modulated by the level of psychopathology, and by the patients mentalization and signal detection ability.

## Data Availability

The data supporting the findings of this study are available from the corresponding author upon reasonable request.

## References

[1] Kahn RS, Sommer IE, Murray RM, Meyer-Lindenberg A, Weinberger DR, Cannon TD, et al. Schizophrenia. Nat Rev Dis Primers. 2015;1:15067. 27189524 10.1038/nrdp.2015.67

[2] Correll CU, Schooler NR. Negative symptoms in schizophrenia: a review and clinical guide for recognition, assessment, and treatment. Neuropsychiatr Dis Treat. 2020;16:519–34. 32110026 10.2147/NDT.S225643 PMC7041437

[3] Green MF, Horan WP, Lee J. Social cognition in schizophrenia. Nat Rev Neurosci. 2015;16:620–31. 26373471 10.1038/nrn4005

[4] Green MF, Bearden CE, Cannon TD, Fiske AP, Hellemann GS, Horan WP, et al. Social cognition in schizophrenia, part 1: performance across phase of illness. Schizophr Bull. 2012;38:854–64. 21345917 10.1093/schbul/sbq171 PMC3406534

[5] Barbato M, Liu L, Penn DL, Keefe RS, Perkins DO, Woods SW, et al. Social cognition as a mediator between neurocognition and functional outcome in individuals at clinical high risk for psychosis. Schizophr Res. 2013;150:542–6. 24012459 10.1016/j.schres.2013.08.015 PMC3839963

[6] Sumiyoshi T, Higuchi Y, Itoh T, Matsui M, Arai H, Suzuki M, et al. Effect of perospirone on P300 electrophysiological activity and social cognition in schizophrenia: a three-dimensional analysis with sloreta. Psychiatry Res. 2009;172:180–3. 19386475 10.1016/j.pscychresns.2008.07.005

[7] Riccardi C, Montemagni C, Del FE, Bellino S, Brasso C, Rocca P. Pharmacological treatment for social cognition: current evidence. Int J Mol Sci. 2021;22:7457. 34299076 10.3390/ijms22147457 PMC8307511

[8] Green MF, Horan WP, Lee J. Nonsocial and social cognition in schizophrenia: current evidence and future directions. World Psychiatry. 2019;18:146–61. 31059632 10.1002/wps.20624 PMC6502429

[9] Lee SY, Namkoong K, Cho HH, Song DH, An SK. Reduced visual P300 amplitudes in individuals at ultra-high risk for psychosis and first-episode schizophrenia. Neurosci Lett. 2010;486:156–60. 20858531 10.1016/j.neulet.2010.09.035

[10] Ford JM, Mathalon DH, Kalba S, Marsh L, Pfefferbaum A. N1 and P300 abnormalities in patients with schizophrenia, epilepsy, and epilepsy with schizophrenialike features. Biol Psychiatry. 2001;49:848–60. 11343681 10.1016/s0006-3223(00)01051-9

[11] Czeszumski A, Eustergerling S, Lang A, Menrath D, Gerstenberger M, Schuberth S, et al. Hyperscanning: a valid method to study neural inter-brain underpinnings of social interaction. Front Hum Neurosci. 2020;14:39. 32180710 10.3389/fnhum.2020.00039 PMC7059252

[12] Barde A, Gumilar I, Hayati AF, Dey A, Lee G, Billinghurst M. A review of hyperscanning and its use in virtual environments. Informatics. 2020;7:55.

[13] Montague PR, Berns GS, Cohen JD, McClure SM, Pagnoni G, Dhamala M, et al. Hyperscanning: simultaneous fMRI during linked social interactions. Neuroimage. 2002;16:1159–64. 12202103 10.1006/nimg.2002.1150

[14] Hirata M, Ikeda T, Kikuchi M, Kimura T, Hiraishi H, Yoshimura Y, et al. Hyperscanning MEG for understanding mother-child cerebral interactions. Front Hum Neurosci. 2014;8:118. 24624076 10.3389/fnhum.2014.00118 PMC3941301

[15] Jeon YW, Polich J. Meta-analysis of P300 and schizophrenia: patients, paradigms, and practical implications. Psychophysiology. 2003;40:684–701. 14696723 10.1111/1469-8986.00070

[16] Bramon E, Rabe-Hesketh S, Sham P, Murray RM, Frangou S. Meta-analysis of the P300 and P50 waveforms in schizophrenia. Schizophr Res. 2004;70:315–29. 15329307 10.1016/j.schres.2004.01.004

[17] Smith JL, Barry RJ, Steiner GZ. CNV resolution does not cause NoGo anteriorisation of the P3: a failure to replicate Simson et al. Int J Psychophysiol. 2013;89:349–57. 23669175 10.1016/j.ijpsycho.2013.05.002

[18] Pomarol-Clotet E, Oh TM, Laws KR, McKenna PJ. Semantic priming in schizophrenia: systematic review and meta-analysis. Br J Psychiatry. 2008;192:92–97. 18245021 10.1192/bjp.bp.106.032102

[19] Wada M, Kurose S, Miyazaki T, Nakajima S, Masuda F, Mimura Y, et al. The P300 event-related potential in bipolar disorder: a systematic review and meta-analysis. J Affect Disord. 2019;256:234–49. 31200163 10.1016/j.jad.2019.06.010

[20] Raggi A, Lanza G, Ferri R. A review on P300 in obsessive-compulsive disorder. Front Psychiatry. 2021;12:751215. 34887786 10.3389/fpsyt.2021.751215 PMC8649722

[21] Hamilton HK, Roach BJ, Bachman PM, Belger A, Carrion RE, Duncan E, et al. Association between P300 responses to auditory oddball stimuli and clinical outcomes in the psychosis risk syndrome. JAMA Psychiatry. 2019;76:1187–97. 31389974 10.1001/jamapsychiatry.2019.2135 PMC6686970

[22] Sumich A, Kumari V, Dodd P, Ettinger U, Hughes C, Zachariah E, et al. N100 and P300 amplitude to Go and No-Go variants of the auditory oddball in siblings discordant for schizophrenia. Schizophr Res. 2008;98:265–77. 18022352 10.1016/j.schres.2007.09.018

[23] Solis-Vivanco R, Mondragon-Maya A, Reyes-Madrigal F, Fuente-Sandoval C. Impairment of novelty-related theta oscillations and P3a in never medicated first-episode psychosis patients. NPJ Schizophr. 2021;7:15. 33637757 10.1038/s41537-021-00146-3 PMC7910533

[24] Kaur M, Battisti RA, Ward PB, Ahmed A, Hickie IB, Hermens DF. MMN/P3a deficits in first episode psychosis: comparing schizophrenia-spectrum and affective-spectrum subgroups. Schizophr Res. 2011;130:203–9. 21550211 10.1016/j.schres.2011.03.025

[25] Weisbrod M, Kiefer M, Marzinzik F, Spitzer M. Executive control is disturbed in schizophrenia: evidence from event-related potentials in a Go/NoGo task. Biol Psychiatry. 2000;47:51–60. 10650449 10.1016/s0006-3223(99)00218-8

[26] Fukuda M, Niwa S, Hiramatsu K, Hata A, Saitoh O, Hayashida S, et al. Behavioral and P3 amplitude enhancement in schizophrenia following feedback training. Schizophr Res. 1997;25:231–42. 9264178 10.1016/s0920-9964(97)00028-5

[27] de la Asuncion J, Bervoets C, Morrens M, Sabbe B, De Bruijn ER. EEG correlates of impaired self-other integration during joint-task performance in schizophrenia. Soc Cogn Affect Neurosci. 2015;10:1365–72. 25759471 10.1093/scan/nsv023 PMC4590534

[28] Deng F, Bueber MA, Cao Y, Tang J, Bai X, Cho Y, et al. Assessing social cognition in patients with schizophrenia and healthy controls using the reading the mind in the eyes test (RMET): a systematic review and meta-regression. Psychol Med. 2024;54:847–73. 38173096 10.1017/S0033291723003501 PMC13285861

[29] Dehelean L, Romosan AM, Bucatos BO, Papava I, Balint R, Bortun AMC, et al. Social and neurocognitive deficits in remitted patients with schizophrenia, schizoaffective and bipolar disorder. Healthcare. 2021;9:365. 33805007 10.3390/healthcare9040365 PMC8063917

[30] Tsoi DT, Lee KH, Khokhar WA, Mir NU, Swalli JS, Gee KA, et al. Is facial emotion recognition impairment in schizophrenia identical for different emotions? a signal detection analysis. Schizophr Res. 2008;99:263–9. 18180142 10.1016/j.schres.2007.11.006

[31] American Psychiatric Association, DSM-5 Task Force. Diagnostic and statistical manual of mental disorders: DSM-5™. 5th ed. American Psychiatric Publishing, Inc; 2013.

[32] Derogatis LR, Cleary PA. Factorial invariance across gender for the primary symptom dimensions of the SCL-90. Br J Soc Clin Psychol. 1977;16:347–56. 588890 10.1111/j.2044-8260.1977.tb00241.x

[33] Unoka Z, Rozsa S, Ko N, Kallai J, Fabian A, Simon L. Validity and reliability of the SCL-90 in a Hungarian population sample. Psychiatr Hung. 2004;19:235–43.

[34] Langan J, Martin D, Shajahan P, Smith DJ. Antipsychotic dose escalation as a trigger for neuroleptic malignant syndrome (NMS): literature review and case series report. BMC Psychiatry. 2012;12:214. 23194104 10.1186/1471-244X-12-214 PMC3546951

[35] Kay SR, Fiszbein A, Opler LA. The positive and negative syndrome scale (PANSS) for schizophrenia. Schizophr Bull. 1987;13:261–76. 3616518 10.1093/schbul/13.2.261

[36] Marder SR, Davis JM, Chouinard G. The effects of risperidone on the five dimensions of schizophrenia derived by factor analysis: combined results of the North American trials. J Clin Psychiatry. 1997;58:538–46. 9448657 10.4088/jcp.v58n1205

[37] Hopkins SC, Ogirala A, Loebel A, Koblan K. S.Transformed PANSS factors intended to reduce pseudospecificity among symptom domains and enhance understanding of symptom change in antipsychotic-treated patients with schizophrenia. Schizophr Bull. 2018;44:593–602. 28981857 10.1093/schbul/sbx101 PMC5890480

[38] Lang PJ, Bradley MM, Cuthbert BN. International affective picture system (IAPS): affective ratings of pictures and instruction manual. In: Technical Report A-6. Gainesville: University of Florida; 2005.

[39] Lang PJ, Bradley MM, Cuthbert BN. International affective picture system (IAPS): affective ratings of pictures and instruction manual. In: Techical Report A-8. Gainesville, FL: University of Florida; 2008.

[40] Barraza P, Dumas G, Liu H, Blanco-Gomez G, van den Heuvel MI, Baart M, et al. Implementing EEG hyperscanning setups. MethodsX. 2019;6:428–36. 30906698 10.1016/j.mex.2019.02.021 PMC6411510

[41] Baron-Cohen S, Jolliffe T, Mortimore C, Robertson M. Another advanced test of theory of mind: evidence from very high functioning adults with autism or asperger syndrome. J Child Psychol Psychiatry. 1997;38:813–22. 9363580 10.1111/j.1469-7610.1997.tb01599.x

[42] Baron-Cohen S, Wheelwright S, Hill J, Raste Y, Plumb I. The “Reading the Mind in the Eyes” test revised version: a study with normal adults, and adults with Asperger syndrome or high-functioning autism. J Child Psychol Psychiatry. 2001;42:241–51. 11280420

[43] Makowski D. The psycho package: an efficient and publishing-oriented workflow for psychological science. J Open Source Softw. 2018;3:470.

[44] Knežević M, Marinković K. Neurodynamic correlates of response inhibition from emerging to mid adulthood. Cogn Dev. 2017;43:106–18. 29081593 10.1016/j.cogdev.2017.03.002 PMC5658138

[45] De Sanctis P, Foxe JJ, Czobor P, Wylie GR, Kamiel SM, Huening J, et al. Early sensory-perceptual processing deficits for affectively valenced inputs are more pronounced in schizophrenia patients with a history of violence than in their non-violent peers. Soc Cogn Affect Neurosci. 2013;8:678–87. 22563006 10.1093/scan/nss052 PMC3739916

[46] Bryk, AS, & Raudenbush, SW. Hierarchical linear models: applications and data analysis methods. American Psychological Association; Newbury Park, CA: Sage Publications 1992.

[47] Gibbons RD, Hedeker D, Waternaux C, Davis JM. Random regression models: a comprehensive approach to the analysis of longitudinal psychiatric data. Psychopharmacol Bull. 1988;24:438–43. 3153505

[48] Benjamini Y, Drai D, Elmer G, Kafkafi N, Golani I. Controlling the false discovery rate in behavior genetics research. Behav Brain Res. 2001;125:279–84. 11682119 10.1016/s0166-4328(01)00297-2

[49] Dumas G. From inter-brain connectivity to inter-personal psychiatry. World Psychiatry. 2022;21:214–5. 10.1002/wps.20987. 35524605 10.1002/wps.20987 PMC9077612

[50] Kim SW, Moon SY, Hwang WJ, Lho SK, Oh S, Lee TY, et al. Impaired performance on the reading the mind in the eyes test in first-episode psychosis and clinical high risk for psychosis. Psychiatry Investig. 2020;17:1200–6. 33301666 10.30773/pi.2020.0264 PMC8560336

[51] Alvarez R, Velthorst E, Pinkham A, Ludwig KA, Alamansa J, Gaigg SB, et al. Reading the mind in the eyes and cognitive ability in schizophrenia- and autism spectrum disorders. Psychol Med. 2023;53:7913–22. 37522512 10.1017/S0033291723002052 PMC10755246

[52] Chhabra H, Sowmya S, Sreeraj VS, Kalmady SV, Shivakumar V, Amaresha AC, et al. Auditory false perception in schizophrenia: development and validation of auditory signal detection task. Asian J Psychiatr. 2016;24:23–27. 27931901 10.1016/j.ajp.2016.08.006

[53] Williams LE, Blackford JU, Luksik A, Gauthier I, Heckers S. Reduced habituation in patients with schizophrenia. Schizophr Res. 2013;151:124–32. 24200419 10.1016/j.schres.2013.10.017 PMC3908315

[54] Kapur S. Psychosis as a state of aberrant salience: a framework linking biology, phenomenology, and pharmacology in schizophrenia. Am J Psychiatry. 2003;160:13–23. 12505794 10.1176/appi.ajp.160.1.13

[55] Taylor SF, Phan KL, Britton JC, Liberzon I. Neural response to emotional salience in schizophrenia. Neuropsychopharmacology. 2005;30:984–95. 15689961 10.1038/sj.npp.1300679

[56] Murray GK, Corlett PR, Clark L, Pessiglione M, Blackwell AD, Honey G, et al. Substantia nigra/ventral tegmental reward prediction error disruption in psychosis. Mol Psychiatry. 2008;13:239–76. 17684497 10.1038/sj.mp.4002058 PMC2564111

[57] Tripathi A, Kar SK, Shukla R. Cognitive deficits in schizophrenia: understanding the biological correlates and remediation strategies. Clin Psychopharmacol Neurosci. 2018;16:7–17. 29397662 10.9758/cpn.2018.16.1.7 PMC5810454

[58] Almeida VN, Radanovic M. Semantic priming and neurobiology in schizophrenia: a theoretical review. Neuropsychologia. 2021;163:108058. 34655651 10.1016/j.neuropsychologia.2021.108058

[59] Kim HK, Blumberger DM, Daskalakis ZJ. Neurophysiological biomarkers in schizophrenia-P50, mismatch negativity, and TMS-EMG and TMS-EEG. Front Psychiatry. 2020;11:795. 32848953 10.3389/fpsyt.2020.00795 PMC7426515

[60] Umbricht D, Javitt D, Novak G, Bates J, Pollack S, Lieberman J, et al. Effects of clozapine on auditory event-related potentials in schizophrenia. Biol Psychiatry. 1998;44:716–25. 9798075 10.1016/s0006-3223(97)00524-6

[61] Gonul AS, Suer C, Coburn K, Ozesmi C, Oguz A, Yilmaz A. Effects of olanzapine on auditory P300 in schizophrenia. Prog Neuropsychopharmacol Biol Psychiatry. 2003;27:173–7. 12551741 10.1016/s0278-5846(02)00349-4

